# Role of Selected Circulating Tumor Biomarkers in Patients with Skeletal Metastatic Pancreatic Neuroendocrine Neoplasms

**DOI:** 10.3390/jcm12144687

**Published:** 2023-07-14

**Authors:** Violetta Rosiek, Ksenia Janas, Magdalena Witkowska, Beata Kos-Kudła

**Affiliations:** 1 Department of Endocrinology and Neuroendocrine Tumours, Department of Pathophysiology and Endocrinology, Medical University of Silesia, 40-014 Katowice, Poland; 2 Department of Endocrinology and Neuroendocrine Tumours, Medical University of Silesia, 40-014 Katowice, Poland

**Keywords:** pancreatic neuroendocrine neoplasms, bone metastasis, biomarker

## Abstract

We investigated the diagnostic capacity of selected circulating biomarkers (CBMs) for the early detection of bone metastasis (BMets) in patients with pancreatic neuroendocrine neoplasms (PanNENs). A total of 115 patients with PanNENs and 40 controls were enrolled. We measured the serum levels of ferritin, cytokeratin 18 (CY18), CA19-9, CA125, AFP, CEA, and beta-2 microglobulin (B2M). A total of eight PanNEN patients developed BMets, and one hundred seven remained BMets-free. We observed a significantly higher level of CA125 and CY18 in BMets patients vs. non-BMets patients (*p* = 0.01 and *p* = 0.04, respectively). CA125, CY18, and B2M area under receiver operator characteristic (AUROC) analyses differentiated both patients groups; CA125 area under the curve (AUC) 0.77, *p* < 0.01; CY18 AUC data were 0.72, *p* = 0.03, and B2M AUC 0.67, *p* = 0.02. On the basis of CBM metrics in both subgroups, we reached a sensitivity/specificity for CA125 of 75/76%; for CY18 of 75/69%, for B2M of 100/50%, for CA125, and the CY18 combination of 93/90%, respectively. According to current results, CA125 and CY18 seem to have the potential capacity as fair biomarkers for BMets detection, despite the small number of cases. Further studies are warranted in the larger PanNEN patient group.

## 1. Introduction

Neuroendocrine neoplasms (NENs) of the pancreas constitute about 30% of all gastro–entero–pancreatic neuroendocrine neoplasms (GEP-NENs) and 1–2% of all pancreatic tumors [1]. These tumors can be functional pancreatic neuroendocrine neoplasms (F-PanNENs) or non-functional pancreatic neuroendocrine neoplasms (NF-PanNENs) (60–90%). According to the 5th edition of the World Health Organization (WHO) gastrointestinal system classification (2019), these neoplasms are divided into well-differentiated neuroendocrine tumors (NETs) and poorly differentiated neuroendocrine carcinomas (NECs). Additionally, these PanNETs are classified into three subtypes based on the grade of their histological maturity; NET G1—high grade, NET G2—intermediate grade, and NET G3—low grade (according to the number of figures of division and the proliferation index Ki-67).

Over one-third of patients with pancreatic NENs (PanNENs) present with metastatic disease at diagnosis [2]. The 5-year survival rate of PanNENs, for the most part, is associated with distant metastasis [3]. Metastases are present mainly in the liver; however, bone metastases are detected in less than 15% of all NEN patients [4] and in only 4% of pancreatic NEN (PanNEN) patients [4,5]. Bone metastases may be asymptomatic and incidentally detected; therefore, both functional imaging, such as [^68^Ga]Ga-somatostatin analog (SSA) positron emission tomography (PET)/computed tomography (CT)/[^18^F]F-FDG PET/CT, and anatomical scans, such as CT/magnetic resonance imaging (MRI) are needed to assess the disease status of PanNEN [6]. The asymptomatic nature of bone metastases results in an underestimation of the incidence of real bone metastases in NEN patients. The most common symptoms of bone metastases are pain, pathological fractures, and metastatic spinal cord compression. They can lead to malignant hypercalcemia and worsened quality of life [7].

Metastatic disease is always connected with a limited prognosis [8]. Therefore it is essential to find new markers that can predict the probability of metastasis and improve the clinical outcome with accurate treatment. Early detection techniques and treatments for bone metastases, such as bisphosphonate, denosumab, as well as radiation therapy, can significantly reduce the risk of spinal cord compression and pathological fractures, mitigate pain, and thus improve quality of life [9].

Some studies suggest that the presence of certain circulating biomarkers can be useful in the early diagnosis/detection of bone metastases [9,10]. To diagnose bone metastases in PanNEN patients, circulating biomarkers, including ferritin, cytokeratin 18 (CK18), CA19-9, CA125, AFP, CEA, and B2M, were evaluated.

Ferritin is a globular protein, which is synthesized in the liver, spleen, and numerous other body tissues and represents total iron storage in the body. Ferritin can play a role in the angiogenesis, proliferation, and immunosuppression of cancer cells [11]. Unfortunately, the higher level of ferritin correlate with increased disease aggressiveness and worse response to treatment. Additionally, ferritin, through the immune system expression of tumor-associated macrophages, leads to an elevated risk of tumor progression and resistance to treatment [12,13]. In the case of cancer, a higher level of ferritin can also indicate residual neoplastic tissue [14]). CK18 is a structural protein involved in regulating cell growth, apoptosis, mitosis, cancer-related signaling, motility, and many other important processes [15]. It is widely expressed in epithelial tissues of many organs (kidneys, lungs, liver, pancreas, gastrointestinal tract, or mammary gland). Moreover, it is continuously expressed in various cancer tissues and is considered a marker of apoptosis [15,16]. Progression of epithelial tumors is associated with cell apoptosis and increased serum CK18 levels. Studies showed elevated circulating caspase-cleaved cytokeratin 18 in colorectal cancer with liver metastases and hepatocellular carcinoma (HCC) patients. However, in colorectal cancer, levels were significantly higher in patients with higher tumor load and correlated with metastatic volume [16]. Another study suggests that HCC releases CK18 via apoptosis, and HCC patients with low serum CK18 levels have a longer rate of survival [17].

CA19-9 is a cell-surface glycoprotein complex produced by human pancreatic, biliary ductal, gastric, and colon cells. The increased level of CA19-9 may occur in several benign gastrointestinal diseases, but the plasmatic level is severely elevated in pancreatic, biliary, and gastrointestinal cancers [18]. CEA is a non-specific serum marker that functions as a prognostic factor and may monitor the therapy of many neoplasms, such as gastrointestinal carcinomas and lung, breast, pancreatic, and colorectal cancers. The constant increase in CEA levels is usually associated with disease progression, local or distant recurrence.

CA19-9 and CEA are the neoplastic markers assessed mainly in pancreatic cancer (they are increased in 75–85% of pancreatic cancer). CEA sensitivity is far superior to that of CA19-9; however, an increased concentration of CA19-9 is a poor prognostic factor [19]).

CA125 comes under the mucin family of proteins and is a serum tumor marker for multiple cancers, such as ovarian, endometrial, pancreatic, or bladder. It is used to detect the recurrence of the disease, the response to the treatment, and to differentiate malignant and benign lesions [20]. Recent data showed that the serum level of CA125 also correlates with survival in lung cancer [21]. CA125 is expressed on the cell membrane and is unable to penetrate the blood. The membrane damage caused by a.o. inflammation may lead to the elevation of serum CA125 levels [22].

AFP is a glycoprotein produced during embryonic development. In non-pregnant adults, it is present in low serum concentrations, which may be increased in patients with liver, testis, or ovarian cancer. The determination of this marker is important in the management of patients with suspected or diagnosed cancer of the liver, testis, or ovary [23,24]. B2M is a small molecular weight protein ordinarily present on the surface of all nucleated cells, and it forms the light chain in the human leukocyte antigen [25]. Membrane B2M performs multiple immune functions, while serum B2M is a marker of disease severity in renal injury, infections, amyloidosis, aging-related diseases, and lymphoproliferative disorders [26].

This study aimed to assess the efficacy of various circulating biomarkers in the detection of bone metastases in patients with PanNENs. The early detection of bone metastases is crucial to prevent pathological fractures and physical disability in patients with PanNENs and improves prognosis and quality of life. In case of elevated circulating biomarkers levels, the diagnostic procedure and treatment protocol should be changed: shorter intervals between clinical check-ups and imaging scans and more aggressive treatment at earlier stages of the disease.

## 2. Materials and Methods

### 2.1. Study Participants

This study group comprised 115 patients with PanNEN, while the control group consisted of 40 healthy volunteers. The mean age (and range) of the patients in this study group was 53 (19–79), and 50 (25–78) in the control group. The controls were healthy volunteers recruited from the hospital and outpatient clinic personnel. The main inclusion criterion for the patient’s group was confirmed histopathological diagnosis of PanNENs according to the WHOs 2019 classification and the American Joint Committee on Cancer/Union for International Cancer Control’s 2017 type and signed consent to participate in this study. All patients with PanNEN were recruited at the Department of Endocrinology and Neuroendocrine Tumors, Medical University of Silesia, ENETS Neuroendocrine Tumor Center of Excellence.

Exclusion criteria for studied subjects were: age less than 18, pregnancy, renal, liver or heart insufficiency. The local Ethics Committee approved this study. Information on age, sex, body mass index (BMI), level of chromogranin A, 5-hydroxyindole acetic acid, serotonin, grade, clinical stage, and bone metastasis of the patients with PanNEN was assessed through patients’ hospital records. The characteristics of the studied groups are presented in Table 1.

Radiological images were reviewed by two independent operators with a huge experience with neuroendocrine neoplasm—a specialist in radiology (if using CT) or a specialist in nuclear medicine (if using PET/CT scan). For the detection of bone metastases in the majority of patients with PanNEN, we performed a functional examination using [^68^Ga]Ga-DOTATATE PET/CT ([^18^F]F-FDG PET/CT. This was performed mainly for poorly differentiated pancreatic neuroendocrine carcinoma (PanNEC).

In 8 patients with PanNEN (3 men and 5 women), bone metastases were confirmed: using CT in 3 cases, [^68^Ga]Ga-DOTATATE PET/CT in 4 cases, and [^18^F]FDG PET/CT in 1 case.

This study was conducted in accordance with good clinical practice guidelines and the Declaration of Helsinki.

### 2.2. Circulating Biomarkers Measurement

The levels of selected biomarkers in the blood serum are described below. The peripheral blood samples (5 mL) were taken from all study participants, leaving the blood to clot. Blood samples from PanNEN patients were taken at different disease stages: before (2 cases) or after tumor-specific treatment (6 cases). Then, these samples were spun, and next, serum was put into boxes and kept at −80 °C for further analysis.

Enzyme–Linked–Immunosorbent Assay (ELISA) or Enzyme–Immunoassay (EIA) was performed with commercially available kits: ELISA kits for B2M, CY18, and ferritin, and EIA kits for AFP, CA125, CA19-9, and CEA. All immunoassays were conducted at the local laboratory in the Department of Endocrinology and Pathophysiology in Zabrze, Medical University of Silesia in Katowice, adapting the manual protocols described by the producers.

The following biomarker tests were used:

- For ferritin: FERRITIN ELISA, DiaMetra S.r.l. Headquater, SEGRATE (Mi), Italy (catalog number DKO039); reference ranges were 20–400 ng/mL for men and 6–350 ng/mL for women; intra-assay precision and inter-assay precision were ≤7.5% and ≤6.1%, respectively.
- For CY18: TPS ELISA, iDL Biotech AB, Bromma, Sweden (catalog number 10-212), the measuring range was 10–1200 U/L, the normal range was <80 U/L, and the detection limit was <6 U/L.
- For CA125: CanAg CA125 EIA, Fujirebio Diagnostics AB, Goteburg, Sweden (catalog number 400-10), the measuring range was 1.5–500 U/mL, the reference range was 5–39 U/mL, the detection limit was <1.5 U/mL, and the intra-assay precision and inter-assay precision were 2.9–4.4% and 3.1–4.0%, respectively.
- For AFP: CanAg AFP EIA, Fujirebio Diagnostics AB, Goteburg, Sweden (catalog number 600-10), the measuring range was 0.5–500 µg/L, the reference range was 0.1–10 µg/L, the detection limit was <0.5 µg/L, and the intra-assay precision and inter-assay precision were 1.6–2.0% and 1.4–2.0%, respectively.
- For CEA: CanAg CEA EIA, Fujirebio Diagnostics AB, Goteburg, Sweden (catalog number 401-10), the measuring range was 0.25–75 µg/L. the reference range was 0.5–9.1 µg/L, the detection limit was <0.25 µg/L, and the intra-assay precision and inter-assay precision were 2.1–2.7% and 1.5–2.7%, respectively.
- For CA19-9: CanAg CA19-9 EIA, Fujirebio Diagnostics AB, Goteburg, Sweden (catalog number 120010), the measuring range was 1–240 U/mL, the reference range was 0–25 U/mL, the detection limit of the assay was <1 U/mL, and the intra-assay precision and inter-assay precision were 3.3–4.5% and 6.2–7.0%, respectively;
- For B2M: β_2_-Microglobulin ELISA, Immunodiagnostic AG, Bensheim, Germany (catalog number K 6210), the reference range was <2.5 mg/L, and the detection limit of the assay was <0.1 mg/L.

### 2.3. Statistical Analysis

Data were presented as the median and interquartile range. The comparison of circulating biomarkers concentrations between study and control groups and patients with PanNEN with and without bone metastases was performed using a nonparametric, 2-tailed Mann–Whitney U test. To investigate the diagnostic capacity of circulating biomarkers in detecting bone metastases, receiver operating characteristic (ROC) curves were plotted, and the area under the curve (AUC), sensitivity, and specificity were calculated. The correlation coefficients between circulating biomarkers concentration, age, BMI, and Ki-67 proliferation index were calculated using the Spearman rank correlation test. The significance threshold in all tests was set at a value of ≤0.05. Statistical analysis was performed using Statistica v. 13.36.0 (StatSoft, Kraków, Poland) software.

## 3. Results

### 3.1. Patients with Pancreatic Neuroendocrine Neoplasms vs. Controls

We present the demographic and clinical characteristics of the participants recruited for this study (PanNEN patients and controls) in Table 1. One hundred and fifteen PanNEN patients were recruited, comprising 43% males and 57% females. In contrast to the control subject group, where the proportion of women significantly dominated (77.5%). Most patients (93%) were diagnosed with well-differentiated NET: fifty-two patients had NET G1, while forty-five patients had NET G2. Only seven percent of these patients (8/115) had bone metastases. Bone metastases were identified at different time points, but these were always secondary metastases following liver or lymph node metastases. Comparisons of the studied circulating biomarkers in patients with pancreatic neuroendocrine neoplasms and controls are presented in Tables S1 and S2 in the Supplementary Materials. Serum CY18, ferritin, CA19-9, CEA, and B2M concentrations in PanNEN patients were significantly higher than in control individuals (*p* < 0.05). The highest AUROC for differentiating PanNENs from controls (>0.7) had CY18, CA19-9, and ferritin (*p* < 0.001), which indicates they are fair biomarkers for PanNEN diagnosis. CEA and BMG could also differentiate PanNENs from controls (*p* < 0.05), but AUC < 0.6 indicates poor diagnostic markers.

The pattern of bone metastasis and clinical characteristics of the patients with pancreatic neuroendocrine neoplasms are shown in Table 2.

### 3.2. Patients with Pancreatic Neuroendocrine Neoplasm, Bone Metastases and Tumor Biomarkers

**Figure 1:**
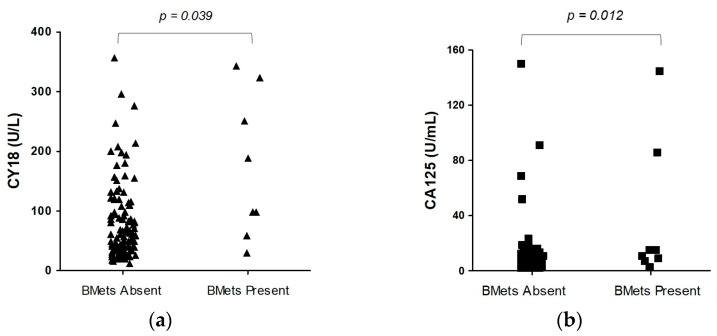
Serum CY18 and CA125 levels in patients with pancreatic neuroendocrine neoplasm (PanNEN): (**a**) Comparison of serum CY18 between PanNEN patients with bone metastasis (BMets) versus PanNEN without BMets (*p* = 0.04); (**b**) Comparison of serum CA125 between PanNEN with BMets versus PanNEN without BMets (*p* = 0.01). Abbreviations: CY18, cytokeratin 18; CA125, cancer antigen 125.

In the second part of this study, we established circulating biomarkers levels according to the presence or absence of bone metastases (Table 2). Before circulating biomarker measurements, all PanNEN patients displayed normal routine lab tests, including alkaline phosphatase, ALT/AST, calcium, or phosphate levels, and neuroendocrine tumor marker levels (chromogranin A, serotonin and 5-hydroxyindole acetic acid). The medians of two circulating biomarkers (CY18 and CA125) in PanNEN patients with bone metastases (*n* = 8) were significantly increased (*p* < 0.05) versus those without bone metastases (*n* = 107). The circulating CY18 level in bone metastatic patients (174.20 U/L ± 121.14; 144 [79–288]) was significantly higher (*p* = 0.04) compared to PanNEN patients without bone metastases (94.17 U/L ± 93.58; 62 [36–120]). The serum CA125 concentration was also elevated (*p* = 0.01) in the first group (36.29 U/mL ± 51.47; n = 13 [8–50]) compared to the second group (9.65 U/mL ± 18.16; n = 6 [3–9]) (Figure 1). The concentrations of other assessed circulating biomarkers, including chromogranin A, serotonin, 5-hydroxyindoleacetic acid, as well as proliferative index Ki-67 and primary tumor size, did not differ significantly between these groups (*p* > 0.05) (Table 3).

### 3.3. Diagnostic Accuracy of Tumor Biomarkers

We calculated the AUC and plotted ROC curves to assess the diagnostic value of circulating biomarkers in bone metastases. Given these analyses, three circulating biomarkers (CA125, CY18, and B2M) could differentiate patients with bone metastases from bone-metastases-free subjects (*p* < 0.05). The accuracy of diagnosis in patients with bone metastases was 75% for CA125 compared to 70% for CY18 and 53% for B2M.

#### 3.3.1. Cancer Antigen 125 (CA125)

The median value of CA125 for the PanNEN patients at the time of bone metastatic disease was 13 U/mL and 6 U/l for those of the non-bone metastatic group (Table 3).

**Figure 2:**
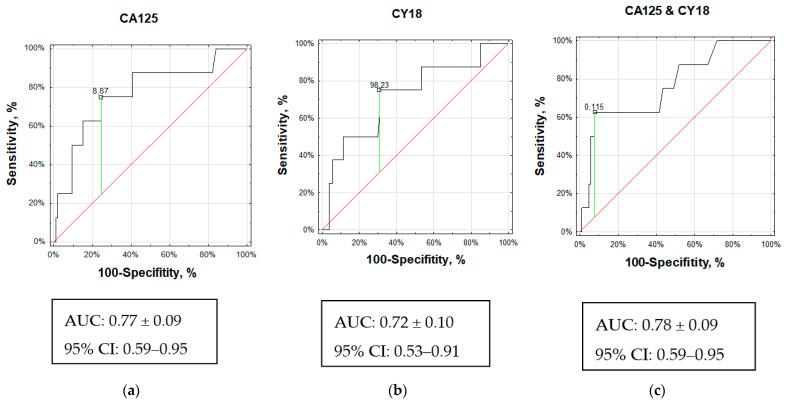
Performance of serum CA125 and CY18 for detecting PanNEN patients with bone metastases (BM-PanNET patients). The receiver operating characteristic (ROC) curves and the area under the curves (AUC) for BM-PanNEN patients versus non-BM-PanNEN patients are displayed: (**a**) Individual ROC curve and AUC for serum CA125 (AUC 0.77, 95% CI 0.59–0.95. *p* < 0.01); (**b**) Individual ROC curve and AUC for serum CY18 (AUC 0.72, 95% CI 0.53–0.91. *p* = 0.03); (**c**) ROC curve and AUC for serum CA125 and CY18 combination (AUC 0.78, 95% CI 0.59–0.95. *p* < 0.01).

The AUC analyses could differentiate PanNEN patients with bone metastases from PanNEN without bone metastases (*p* < 0.01, AUC 0.77 ± 0.09; z score: 2.87, Youden index J: 50%). It should be noted that an AUC of 0.77 would be considered a useful biomarker of bone metastases (Figure 2a). For the cut-off value of 8.87 U/mL for CA125, the specificity/sensitivity was 76/75%, and the accuracy was similarly 75%.

#### 3.3.2. Cytokeratin 18 (CY18)

The median value of CY18 was 144 U/L for the PanNEN patients with bone metastases and 62 U/L for those of the non-bone metastatic group (Table 3).

AUC analysis could differentiate PanNEN with bone metastases from PanNEN without bone metastases (*p* = 0.03, AUC 0.72 ± 0.10; z score: 2.22, Youden index J: 44%). It should be noted that an AUC of 0.72 would be considered a fair biomarker of bone metastases (Figure 2b). For the cut-off value of 98.23 U/L for CY18, the accuracy, sensitivity, and specificity were 70%, 75%, and 69%, respectively.

#### 3.3.3. Combination of CY18 and CA125 (multiROC)

Next, we combined the CA125 and CY18 serum levels to construct a further ROC curve. This demonstrated that the serum CA125 and CY18 classifiers had higher accuracy for bone metastases with an AUC similar to CA125 of 0.78 (95% CI 0.59–0.95; Figure 2c). Thus, the combination of CA125 and CY18 in serum was similar to individual CA125 distinguishing between PanNEN with bone metastases and PanNEN without bone metastases (Figure 2c). The sensitivity for the cut-off value of 0.12 was calculated as 63%, and the specificity and accuracy were higher at 93% and 90%, respectively.

The CA125 AUC and CY18 AUC > 0.7 (black curves) indicate they are fair biomarkers for PanNENs with BMets. A maximum AUC = 1 identifies an ideal (perfect) differentiation between these groups. The diagonal red line (AUC = 0.5) corresponds to chance discrimination.

The individual CA125 AUC and combination AUC of CA125 and CY18 were greater than 0.75, which may indicate clinically helpful biomarkers for distinguishing between PanNEN with bone metastases and PanNEN without bone metastases.

#### 3.3.4. Beta-2 Microglobulin (B2M)

The median values of B2M for PanNEN patients with bone metastases and those in the non-bone metastatic group were not significantly different (*p* > 0.05) (Table 3).

Although AUC analyses could differentiate PanNEN with bone metastases from PanNEN without bone metastases (*p* = 0.02), an AUC of 0.67 would be considered a poor biomarker of bone metastases. Youden index J was 50%. The sensitivity and specificity for the cut-off value of 1.16 mg/L were calculated as 100 and 50%, respectively; the accuracy was 53% (Table S3 in Supplementary Materials).

#### 3.3.5. Other Tumor Markers

The median values of other tumor markers (Ferritin, A19-9, AFP, CEA, B2M) for the PanNEN patients with bone metastases and those in the non-bone metastatic group were also not significantly different (*p* > 0.05) (Table 2).

In addition, AUROC analysis of these markers could not differentiate patients with bone metastasis from bone metastasis-free cases (Table S3 in Supplementary Materials).

#### 3.3.6. Neuroendocrine Tumor Markers (Chromogranin A, Serotonin, and 5-Hydroxyinoleacetic Acid)

Based on the AUC and ROC curve analyses of chromogranin A, serotonin, and 5-hydroxyindole acetic acid, we may not differentiate PanNEN with bone metastases from PanNEN without them (*p* > 0.05). The AUC of these tumor markers below 0.6 indicates they are poor predictive markers. These data are presented in Figures S1–S3 in Supplementary Materials.

## 4. Discussion

The most important factor influencing NEN patients’ prognosis is metastasis [3,8]. Most frequently, metastases are located in the liver but can also be found in other organs such as the lungs, brain, or bones [4,5]. The presence of metastasis is always connected with poor prognosis and worse outcomes. Some studies showed that patients with BMets have shorter survival compared to patients with metastasis in other locations [4,9].

We tried to find effective biomarkers that may be useful in the detection of bone metastases in patients with PanNEN. We analyzed potentially valuable proteins such as ferritin, CA19-9, CA125, AFP, CEA, CK18, and B2M. We revealed that levels of three biomarkers (CA125, CY18, and B2M) were significantly higher in patients with metastatic bone disease than those without bone metastases.

Serum cytokeratin (CK) levels are low in healthy individuals. During the process of carcinogenesis, which includes proteolytic degradation in dying cells, abnormal mitosis, and apoptosis, fragments of CKs are released into the blood, and their level is raised [17,27]. As a result, they can be useful as tumor markers and help to predict tumor progression and metastasis formation [27]. Therefore, this study tried to find the correlation between serum CK18 levels and the probability of bone metastases in patients with PanNEN. Cytokeratin 18 exhibits overexpression in many types of cancer originating from epithelial organs [28,29,30]. A study by Menz A. et al. confirmed the appearance of adenocarcinomas of the lung, pancreas, small bowel, prostate, and cervix uteri [28].

Other investigators showed a higher expression of CK18 in Paget’s tumor cells (skin lesions and lymph node metastases). Furthermore, soluble CK18 forms were significantly higher in patients with metastasis compared to non-metastatic disease [31,32].

On the other hand, some studies showed a negative correlation between CK18 concentration and disease advancement (the lower CK18 concentrations were related to lymph node metastasis and poor survival in patients with breast cancer) [33]. A study by Yin B. et al. revealed a negative correlation between serum CK18 level and tumor aggressiveness in prostate cancer [34].

To our knowledge, serum CK18 levels in PanNEN patients with bone metastases were not studied. Our study noted a difference in CK 18 serum levels in patients with and without bone metastases. Patients with bone metastases had a higher level of CK18, so it seems to be clinically useful as a diagnostic factor for bone lesions.

We also tried to find a correlation between the CA125 level and the incidence of bone metastases in patients with PanNEN. Increased CA125 levels can be connected with many malignancies localized in the ovary, breast, liver, lung, pancreas, gastrointestinal tract, uterine, cervix, and endometrium [35]. Its level can also be elevated in healthy individuals such as women in the follicular phase of the menstrual cycle, during pregnancy [35], and in non-malignant conditions such as endometriosis, ovarian cysts, pelvic inflammatory disease, cirrhosis, hepatitis, ascites or heart failure [36,37,38]. CA125 has been used so far as a marker of ovarian cancer. It has limited sensitivity in detecting ovarian cancer, but it helps monitor response to treatment and detect residual or recurrent disease after therapy. Its level also correlates with staging and tumor size [39,40,41]. Zhang M. et al. proved that CA125 is significantly elevated not only in ovarian cancer but also in lung and pancreatic cancer and decreased in rectal cancer [42]. In the current study, the level of CA125 was significantly higher in patients with bone metastases versus patients without bone metastatic disease.

Another correlation we observed in this research is the relation between B2M level and the incidence of bone metastases in patients with PanNEN. B2M is involved in many important biological processes, such as the regulation of survival, proliferation, and apoptosis [43,44]. It also stimulates the growth and progression of several cancers or metastasis in cancer cells. Prizment A. et al. pointed out that higher serum B2M is associated with increased colorectal cancer risk. The authors also suggested a significant association between serum B2M and mortality from total, lung, and hematological cancers [45]. The elevated level of B2M is supposed to be a strong indicator of poor prognosis and reduced survival. In prostate cancer, studies found that advanced prostate cancer is connected with an increase in serum levels of B2M [46,47].

Our analysis also tried to find a link between serum levels of common neuroendocrine tumor markers such as chromogranin A (CgA), serotonin, and 5-hydroxy indoleacetic acid (5-HIAA) and bone metastases in PanNETs.

Results of a study by Tomasetti P et al. indicated the diagnostic value of plasma CgA levels in advanced PanNETs with multiple liver metastases [48]. Another study showed higher levels of CgA in metastatic PanNETs compared to localized disease [49]. This effect was also observed in prostate cancer. Patients diagnosed with metastatic castration-resistant prostatic cancer displayed 2–3 times higher levels of CgA compared to those with localized disease [50]. Serotonin and its primary metabolite—5-HIAA is used in the diagnosing and monitoring of carcinoid tumors, a subset of serotonin-secreting neuroendocrine tumors. Studies have shown a potential stimulatory effect of serotonin on cancer cell proliferation, invasion, dissemination, and tumor angiogenesis [51]. Moreover, some research reported that serotonin exerted complex effects on cytokine release from macrophages and monocytes and hence is a crucial factor in controlling the immune microenvironment and may promote tumorigenesis [52].

The analysis performed in this study showed that CgA, serotonin, and 5-HIAA levels did not have the capacity to function as biomarkers for detecting bone metastasis.

Opposite these findings, in an Italian research study by Sara Massironi et al. [53], the median CgA levels were significantly higher in GEP-NEN patients with metastases than those without metastases. In that study, the authors enrolled a total of 181 GEP-NEN patients, including 81 pancreatic NEN, and have shown the significant prognostic relevance of plasma CgA. Similarly, a meta-analysis by Rossi et coauthors [54] revealed that chromogranin A could prevent a diagnosis of recurrence/progression rather than rule it out. It is more reliable when used to monitor disease progression and for the early detection of recurrence after treatment rather than in the diagnostic setting.

In this study, we also tried to find a relationship between other biomarkers (ferritin, CA 19-9, AFP, CEA) and the incidence of bone metastases in patients with PanNEN. The differences between these groups were not statistically significant, so it is possible that in PanNEN, these biomarkers have no utility for bone metastases detection. The useful circulating biomarkers for patients with bone metastases detection were Ca125, CY18, and B2M. They seem to have the diagnostic capacity as fair single biomarkers for the detection of bone metastases. However, the given circulating biomarker measurement performances can not be considered adequate for clinical decision-making. However, more studies on larger groups are required because of the small proportion of patients with bone metastases.

Current research demonstrated a serum panel of biomarkers (CA125 and CY18) to differentiate PanNEN patients with bone metastases from PanNEN patients without bone metastases with good metrics (AUC of 0.78). Indeed, significantly elevated concentrations of these biomarkers in patients with PanNEN may be useful for confirming the clinical suspicion of bone metastases in cases of diagnostic dilemma (difficulties in CT/MRI scan interpretation).

The use of these markers In clinical practice, in our view, could be helpful in the interpretation of unclear bone lesions or screening for further diagnostic workup.

## 5. Conclusions

It is not possible to draw solid conclusions based on only eight patients with pancreatic neuroendocrine neoplasm with bone metastases. According to current findings, CA125 and CY18 might potentially have the diagnostic capacity as fair single biomarkers for the detection of bone metastases should become despite the small sample size. Further prospective studies are needed in the larger patient group with pancreatic neuroendocrine neoplasm.

## 6. Study Limitations

First, the total sample of patients with bone metastases was relatively small because PanNEN is rare. Thus, we could not determine a predictive and prognostic value of circulating biomarkers for bone metastases.

Second, the majority of the PanNEN patients were treated before the first presentation of bone metastases.

## Figures and Tables

**Table 1:** Clinical characteristics of the study participants.

Variable	Category	PanNEN Patients	Controls
Number	No.	115	40
Age (years)	Mean (range)	53 (19–79)	50 (25–78)
Gender	Males	49 (43%)	9 (23%)
	Females	66 (57%)	31 (77%)
BMI (kg/m^2^)	<30	101 (88%)	N/A
	>30	14 (12%)	
Grade	NET G1	52 (45%)	N/A
	NET G2	45 (39%)	
	NET G3	3 (3%)	
	NEC	5 (4%)	
Clinical stage	I	31 (27%)	N/A
	II	26 (23%)	
	III	14 (12%)	
	IV	44 (38%)	
Bone metastases	Yes	8 (7%)	N/A
	No	107 (93%)	

Abbreviations: BMI, body mass index; N/A, not applicable; NEC, neuroendocrine carcinoma; NET, neuroendocrine tumors; No., number of cases; PanNEN, pancreatic neuroendocrine neoplasms.

**Table 2:** Bone metastasis pattern and clinical characteristics of the patients with pancreatic neuroendocrine neoplasms.

ID	Case 1	Case 2	Case 3	Case 4	Case 5	Case 6	Case 7	Case 8
Sex	Female	Male	Female	Male	Male	Female	Female	Female
Age (year)	64	25	70	33	74	54	42	60
BMI (kg/m^2^)	23.23	21.48	20.78	19.32	29.54	17.31	19.71	27.34
Functional status	NF-PNEN	NF-PNEN	NF-PNEN	NF-PNEN	NF-PNEN	F-PNEN	NF-PNEN	NF-PNEN
Ki-67 (%) of primary	1	10	10	3	3	2	50	60
Grade	NET G1	NET G2	NET G2	NET G2	NET G1	NET G1	NEC	NEC
No. of BM lesion	single	multiple	multiple	multiple	single	multiple	single	single
Localisation of BMets	right pubic bone	vertebrae rib sternum	vertebrae humerus	vertebrae	right rib	vertebrae sacrum	right hip bone	right shoulder blade
Method used for detection of BMets	68Ga PET/CT	CT	68Ga PET/CT	CT	68Ga PET/CT	CT	FDG PET/CT	68Ga PET/CT
Time point of BMets occurrence after initial diagnosis (months)	8	41	16	29	5	3	7	1
Pancreatic primary	body	body	tail	tail	body	tail	head	head
Tumor size (mm)	11	16	43	35	10	83	84	36
Previous type of treatment	surgery	SSA PRRT everolimus CHTH	SSA	CHTH	N/A	N/A	Surgery CHTH RTH	CHTH

Abbreviations: BMets, bone metastasis; BMI, body mass index; CHTH, chemotherapy; CT, computed tomography; [^18^F]FDG PET, 18F-fluorodeoxyglucose Positron Emission Tomography; F-PanNEN, functional pancreatic neuroendocrine neoplasms; ^68^Ga PET, Gallium Positron Emission Tomography; NEC, neuroendocrine carcinoma; NET, neuroendocrine tumors; NF-PanNEN, non-functional pancreatic neuroendocrine neoplasms; N/A, not applicable; No., number of cases; PRRT, peptide receptor radionuclide therapy; RTH, radiotherapy; SSA, somatostatin analogs.

**Table 3:** The comparison between the clinical characteristics and tumor markers levels in PanNEN patients with (BM-PanNEN patients) and without bone metastasis (non-BM-PanNEN patients) (Mann–Whitney U Test).

Variable	Metastatic PanNEN Patients (*n* = 8) Median [IR]	Non-Metastatic PanNEN Patients (*n* = 107) Median [IR]	*p* Value
Age (years)	57 [38–67]	55 [42–65]	NS
BMI (kg/m^2^)	21 [20–25]	25 [23–28]	NS
CY18 (U/L)	144 [79–288]	62 [36–120]	0.04
CA125 (U/mL)	13 [8–50]	6 [3–9]	0.01
Ferritin (ng/mL)	129 [47–194]	73 [28–135]	NS
CA19-9 (U/mL)	15 [4–19]	9 [5–16]	NS
AFP (µg/L)	3 [2–12]	3 [2–5]	NS
CEA (µg/L)	2 [1–5]	1 [1–2]	NS
B2M (mg/L)	1 [1–2]	1 [1–2]	NS
CgA (µg/L)	84 [44–678]	45 [27–98]	NS
serotonin (ng/mL)	200 [169–318]	245 [146–372]	NS
5-HIAA (mg/24 h)	3 [3–3]	3 [2–5]	NS
Ki-67 (%)	7 [2–35]	3 [1–5]	NS
Tumor size (mm)	43 [11–83]	27 [16–47]	NS

Abbreviations: ACC, accuracy; AFP, alpha-fetoprotein; B2M, beta-2 microglobulin; CA125, cancer antigen 125; CA19-9, carbohydrate antigens 19-9; CEA, carcinoembryonic antigen; CgA, chromogranin A; CY18, cytokeratin 18; 5-HIAA, 5-hydroxyindoleacetic acid; IR, interquartile range; Ki-67, proliferation index; NS, not significant; PanNEN, pancreatic neuroendocrine neoplasm.

## Data Availability

All data are available upon any reasonable request.

## Supplementary Materials

The following supporting information can be downloaded at: https://www.mdpi.com/article/10.3390/jcm12144687/s1, Table S1: The comparison of the tumor markers in patients with pancreatic neuroendocrine neoplasms (PanNENs) and controls (Mann–Whitney U Test); Table S2: The serum tumor markers assay metrics in the diagnosis of patients with pancreatic neuroendocrine neoplasm (PanNEN); Table S3: The serum tumor markers assay metrics in the bone metastases detection of patients with pancreatic neuroendocrine neoplasm (PanNEN); Figure S1: The AUROC for chromogranin A (CgA) levels in patients with pancreatic neuroendocrine neoplasm (PanNENs) with and without bone metastasis (BMets); Figure S2: The AUROC for serotonin levels in patients with pancreatic neuroendocrine neoplasm (PanNENs) with and without bone metastasis (BMets); Figure S3: The AUROC for 5-hydroxyindole acetic acid (5-HIAA) levels in patients with pancreatic neuroendocrine neoplasm (PanNENs) with and without bone metastasis (bMets).
