# Supplementary material for: Role of Selected Circulating Tumor Biomarkers in Patients with Skeletal Metastatic Pancreatic Neuroendocrine Neoplasms
Source: J Clin Med. 2023 Jul 14;12(14):4687. doi: 10.3390/jcm12144687 (PMC10380808; doi:10.3390/jcm12144687)
Supplement: Supplementary file 1 [file jcm-12-04687-s001.zip › jcm-2438357-supplementary.pdf]

Journal of
Clinical Medicine

MDPI

Article

Role of Selected Circulating Tumor Biomarkers in Patients with Skeletal Metastatic Pancreatic Neuroendocrine Neoplasms

Violetta Rosiek [1,2,*], Ksenia Janas [1,2], Magdalena Witkowska [1] and Beata Kos-Kudła [1,2]

1 Department of Endocrinology and Neuroendocrine Tumours, Medical University of Silesia, 40-014 Katowice, Poland; ksenia.janas@sum.edu.pl (K.J.); bkoskudla@sum.edu.pl (B.K.-K.); mgd.witkowska@gmail.com (M.W.)
2 Department of Pathophysiology and Endocrinology, Medical University of Silesia, 40-014 Katowice, Poland
* Correspondence: vrosiek@sum.edu.pl

Table S1. The comparison of the tumour markers in patients with pancreatic neuroendocrine neoplasms (PanNENs) and controls (Mann-Whitney U Test).

Variable	PanNEN patients (n=115)	Controls (n=40)	p
	median [interquartile range]		
CY18 (U/L)	64.67 [35.96 - 121.55]	31.51 [23.05 - 45.67]	< 0.01
Ferritin (ng/mL)	75.43 [29.29 - 144.33]	19.19 [8.80 - 94.81]	< 0.01
CA19-9 (U/mL)	9.16 [5.00 - 15.70]	4.10 [2.19 - 7.66]	< 0.01
CA125 (U/mL)	5.99 [3.15 - 9.67]	5.26 [3.37 - 7.95]	NS
AFP (µg/L)	2.97 [1.95 - 4.76]	2.64 [1.74 - 3.75]	NS
CEA (µg/L)	1.13 [0.66 - 2.02]	0.79 [0.49 - 1.51]	0.03
B2M (mg/L)	1.20 [0.91 - 1.59]	0.94 [0.84 - 1.12]	< 0.01
CgA	45.69 [27.29 – 102.92]	77.50 [48.00 - 107.00]	0.04
serotonin	239.36 [146.70 – 367.52]	132.00 [88.00 - 199.50]	< 0.01
5-HIAA	4.50 [4 - 6]	3.28 [2.31 – 4.61]	< 0.01

Abbreviations Abbreviations: PanNEN, pancreatic neuroendocrine neoplasm; CY18, cytokeratin 18; CA125, cancer antigen 125; CA19-9, carbohydrate antigens 19-9; AFP, alpha-fetoprotein; CEA, carcinoembryonic antigen; B2M, beta-2 microglobulin; NS, not significant; CgA, chromogranin A; 5-HIAA, 5-hydroxyindole acetic acid; Ki-67, proliferation index.

Table S2. The serum tumor markers assay metrics in the diagnosis of patients with pancreatic neuroendocrine neoplasm (PanNEN).

Variable	AUC (95% CI)	SE	Z score	p	Youden index J (%)	Cut-off value	Sensitivity (%)	Specificity (%)	ACC (%)
CY18	0.74 (0.65-0.83)	0.05	5.10	< 0.01	43	48.07 U/L	65	77	68
Ferritin	0.70 (0.59-0.80)	0.05	3.72	< 0.01	41	23.75 ng/mL	83	57	77
CA19-9	0.72 (0.63-0.80)	0.04	4.94	< 0.01	36	4.28 U/mL	78	57	73
CA125	0.55 (0.45-0.64)	0.05	0.93	NS	18	10.85 U/mL	20	97	41
AFP	0.56 (0.46-0.66)	0.05	1.10	NS	14	3.82 µg/L	39	75	48
CEA	0.62 (0.52-0.71)	0.05	2.36	0.02	22	1.95 µg/L	27	95	45
B2G	0.67 (0.58-0.75)	0.04	3.74	< 0.01	35	1.02 mg/L	67	67	68
CgA	0.60 (0.52 - 0.68)	0.04	2.33	0.02	28	32.88 ug/L	36	93	54
serotonin	0.75 (0.67 – 0.83)	0.04	6.38	< 0.01	43	153.60 ng/mL	74	69	72
5-HIAA	0.32 (0.24 - 0.41)	0.04	-4.14	< 0.01	5	10.17 mg/24h	5	100	36

Abbreviations: AUC, area under the curve; CI, confidence interval; SE, standard error; ACC, Accuracy; and other abbreviations see Table S1

Table S3. The serum tumor markers assay metrics in the bone metastases detection of patients with pancreatic neuroendocrine neoplasm (PanNEN).

Variable	AUC (95% CI)	SE	Z score	p	Youden index J (%)	Cut-off value	Sensitivity (%)	Specificity (%)	ACC (%)
CY18	0.72 (0.53 – 0.91)	0.10	2.22	0.03	44	98.23 U/L	75	69	70
Ferritin	0.62 (0.43 – 0.82)	0.10	1.24	NS	28	116.40 ng/mL	63	65	65
CA19-9	0.54 (0.30 – 0.77)	0.12	0.30	NS	31	18.50 U/mL	50	81	79
CA125	0.77 (0.45 – 0.64)	0.09	2.87	< 0.01	50	8.87 U/mL	75	76	75
AFP	0.65 (0.47 – 0.84)	0.10	1.62	NS	36	2.29 µg/L	100	36	40
CEA	0.70 (0.47 - 0.92)	0.11	1.71	NS	38	1.95 µg/L	63	76	75
B2G	0.67 (0.53 - 0.81)	0.07	2.32	0.02	50	1.16 mg/L	100	50	53
CgA	0.65 (0.45 – 0.86)	0.11	1.45	NS	36	42.22 ug/L	88	49	51
serotonin	0.46 (0.30 – 0.62)	0.08	-0.47	NS	19	165.70 ng/mL	88	32	36
5-HIAA	0.44 (0.30 - 0.57)	0.07	-0.91	NS	24	2.18 mg/24h	100	24	30

Abbreviations: see Table S1 and Table S2.

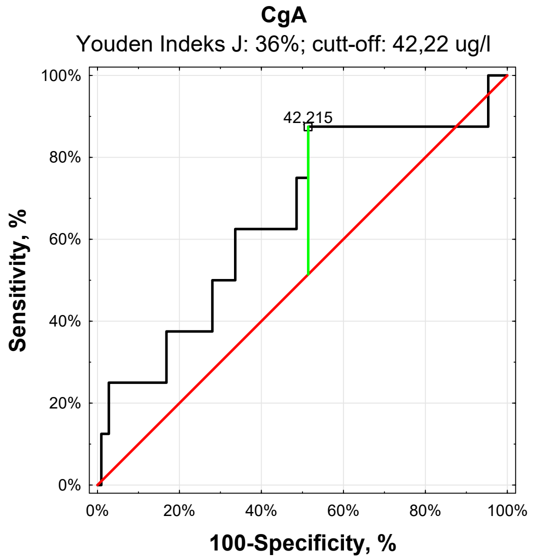

Figure S1. The AUROC for chromogranin A (CgA) levels in patients with pancreatic neuroendocrine neoplasm (PanNENs) with and without bone metastasis (BMets). The AUROC (black curve) for differentiating PanNENs with BMets from PanNENs without BMets was 0.65 (95%CI: 0.45 - 0.86. p = 0.14). A maximum AUC = 1 identifies an ideal (perfect) differentiation between these groups. The diagonal red line (AUC = 0.5) corresponds to chance discrimination. The CgA AUC = 0.65 (black curve) and p = 0.14 indicate that it is a poor biomarker and it can not differentiate PanNENs with BMwts from PanNEN without BMets.

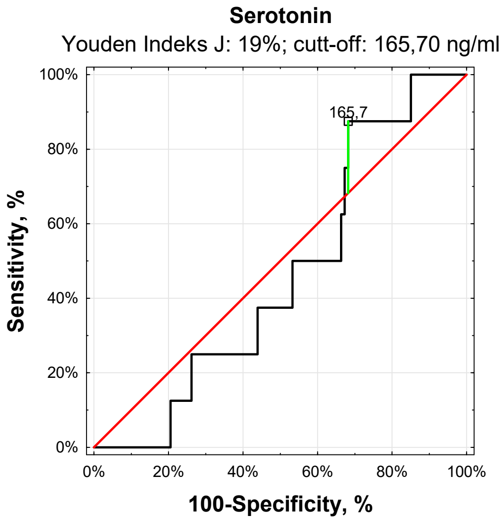

Figure S2. The AUROC for serotonin levels in patients with pancreatic neuroendocrine neoplasm (PanNENs) with and without bone metastasis (BMets). The AUROC (black curve) for differentiating PanNENs with BMets from PanNENs without BMets was 0.46 (95%CI: 0.30 - 0.623. p = 0.64). A maximum AUC = 1 identifies an ideal (perfect) differentiation between these groups. The diagonal red line (AUC = 0.5) corresponds to chance discrimination. The serotonin AUC = 0.46 (black curve) and p = 0.64 indicate that it is a failed biomarker and it can not differentiate PanNENs with BMets from PanNENs without BMets.

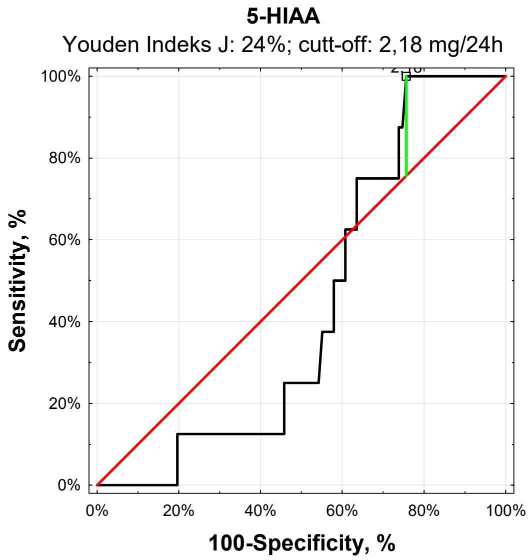

Figure S3. The AUROC for 5-hydroxyindole acetic acid (5-HIAA) levels in patients with pancreatic neuroendocrine neoplasm (PanNENs) with and without bone metastasis (BMets). The AUROC (black curve) for differentiating PanNENs with BMets from PanNENs without BMets was 0.44 (95%CI: 0.30 - 0.57. p = 0.36). A maximum AUC = 1 identifies an ideal (perfect) differentiation between these groups. The diagonal red line (AUC = 0.5) corresponds to chance discrimination. The 5-HIAA AUC = 0.44 (black curve) and p = 0.36 indicate that it is a failed biomarker and it can not differentiate PanNENs with BMets from PanNENs without BMets.
